# Plant-Based Dietary Patterns and Breast Cancer Recurrence and Survival in the Pathways Study

**DOI:** 10.3390/nu13103374

**Published:** 2021-09-25

**Authors:** Ijeamaka C. Anyene, Isaac J. Ergas, Marilyn L. Kwan, Janise M. Roh, Christine B. Ambrosone, Lawrence H. Kushi, Elizabeth M. Cespedes Feliciano

**Affiliations:** 1 Division of Research, Kaiser Permanente Northern California, Oakland, CA 94612, USA; ijeamaka.c.anyene@kp.org (I.C.A.); Isaac.J.Ergas@kp.org (I.J.E.); Marilyn.L.Kwan@kp.org (M.L.K.); Janise.M.Roh@kp.org (J.M.R.); Larry.Kushi@kp.org (L.H.K.); 2 Roswell Park Comprehensive Cancer Center, Buffalo, NY 14263, USA; christine.ambrosone@roswellpark.org

**Keywords:** plant-based diet, dietary patterns, breast cancer, cancer survival, recurrence, lifestyle, survivorship

## Abstract

Plant-based diets are recommended for cancer survivors, but their relationship with breast cancer outcomes has not been examined. We evaluated whether long-term concordance with plant-based diets reduced the risk of recurrence and mortality among a prospective cohort of 3646 women diagnosed with breast cancer from 2005 to 2013. Participants completed food frequency questionnaires at diagnosis and 6-, 25-, and 72-month follow-up, from which we derived plant-based diet indices, including overall (PDI), healthful (hPDI), and unhealthful (uPDI). We observed 461 recurrences and 653 deaths over a median follow-up of 9.51 years. Using multivariable-adjusted Cox proportional hazards models, we estimated hazard ratios (HR) and 95% confidence intervals for breast cancer recurrence and all-cause, breast-cancer-specific, and non-breast-cancer mortality. Increased concordance with hPDI was associated with a reduced hazard of all-cause (HR 0.93, 95% CI: 0.83–1.05) and non-breast-cancer mortality (HR 0.83, 95% CI: 0.71–0.98), whereas increased concordance with uPDI was associated with increased hazards (HR 1.07, 95% CI: 0.96–1.2 and HR 1.20, 95% CI: 1.02–1.41, respectively). No associations with recurrence or breast-cancer-specific mortality were observed. In conclusion, healthful vs. unhealthful plant-based dietary patterns had differing associations with mortality. To enhance overall survival, dietary recommendations for breast cancer patients should emphasize healthful plant foods.

## 1. Introduction

There are an estimated 3.8 million female breast cancer survivors in the United States [1]. Due to insufficient evidence on whether dietary intake influences breast cancer survival and recurrence, breast cancer survivors are encouraged to observe general cancer prevention recommendations [2]. These recommendations include eating a healthy diet with an “emphasis on plant foods”; which is a diet “rich in whole grains, vegetables, fruits and beans” [2,3]. 

Plant-based diets in which individuals consume low amounts of animal-based foods, rather than completely excluding animal-based foods, have been adopted in nutritional research to reflect patterns of eating common in the population. A popular method of assessing plant-based diets is using a dietary pattern index, which is a numerical score measuring concordance to an overall pattern of eating [4]. Many plant-based diet indices, such as the original pro-vegetarian diet score, treat all plant foods the same regardless of quality [5]. To address this shortcoming, plant-based diet indices that differentiate between the consumption of healthful plants (e.g., fruits, whole grains, vegetables, legumes, nuts) and less healthful plants (e.g., refined grains, fruit juices, potatoes, sugar-sweetened beverages (SSBs)) were created [6]. A dietary pattern concordant with a healthful plant-based diet has been associated with reduced risk of coronary heart disease, type 2 diabetes, and breast cancer risk, but to the best of our knowledge, no prior study has examined breast cancer survival, as few cohorts collect dietary assessments both at and following diagnosis, and even fewer measure recurrence or breast-cancer-specific mortality [6,7,8,9,10]. 

Our study examined the relationship between repeated measures of an a priori plant-based diet index and its healthful and unhealthful variations at the time of diagnosis and in the post-diagnosis period, with breast cancer recurrence, all-cause mortality, breast-cancer-specific mortality, and non-breast-cancer mortality in a large cohort of breast cancer survivors.

## 2. Materials and Methods

### 2.1. Study Population

This analysis used data from the Pathways Study, a prospective cohort of 4505 female invasive breast cancer survivors diagnosed at Kaiser Permanente Northern California (KPNC) between the years 2005 and 2013. The protocol for this cohort has been previously published [11]. In brief, participants were enrolled on average 2.3 months (range: 0.7–18.7 months) post-diagnosis and completed an in-person baseline interview. Participants were eligible if they were current KPNC members, at least 21 years of age at the time of diagnosis, but had no previous diagnosis of cancer, spoke English, Spanish, Mandarin, or Cantonese, and lived within a 65-mile radius of a field staffer. 

Dietary data were collected at baseline with a 139-item modified version of the Block 2005 Food Frequency Questionnaire (FFQ) [11]. During follow-up, repeated dietary intake data were collected via mailed questionnaires at 6, 24, and 72 months. In every questionnaire, participants reported how often, on average, did they eat each food in the past 6 months, and how much did they usually eat of the food. NutritionQuest scanned the questionnaires using a nutrient database developed from the USDA Food and Nutrient Database for Dietary Studies. Participants were excluded from this analysis for not completing a dietary assessment at baseline (*n* = 782, 17.4%), reporting a daily total energy intake of less than 400 or greater than 4000 kcal (*n* = 63, 1.4%). An additional 14 (0.3%) participants were excluded for missing data on covariates. This brought the final study analytic population to 3646 women with breast cancer. Of the 3646 participants who had a baseline dietary assessment, 66.1% (*n* = 2410) had a repeat dietary measurement at 6 months, 42.8% (*n* = 1561) at 24 months, and 6.8% (*n* = 247) at 72 months. 

### 2.2. Plant-Based Diet Indices

Using a methodology defined by Satija et al., three plant-based diet indices were created based on the FFQ: an overall plant-based diet index (PDI), a healthful plant-based diet index (hPDI), and an unhealthful plant-based diet index (uPDI) [7]. Table S1 provides an example food item for each food group and indicates the scoring methodology by index. The indices were derived using 18 food groups, where a participant’s total serving size consumption for each food group was broken into cohort-specific quintiles, and each quintile was given a score between 1 and 5. The 18 food groups are made up of healthful plant foods (whole grains, fruits, vegetables, nuts, legumes, vegetable oils, tea, and coffee), unhealthful plant foods (fruit juices, refined grains, potatoes, sugar-sweetened beverages (SSBs), sweets and desserts), and animal foods (dairy, animal fat, egg, meat, fish or seafood, and miscellaneous animal-based foods). Then, from these three main categories, the indices of PDI, hPDI, and uPDI are created. For all three indices, reverse scores are assigned to animal foods. For PDI, positive scores are assigned to all plant foods. For hPDI, positive scores are assigned to healthful plant foods, and reverse scores are assigned to unhealthful plant foods. For uPDI, positive scores are assigned to unhealthful plant foods, and reverse scores are assigned to healthful plant foods. Depending on the index, positive or reverse scores were given when a participant did not consume any foods within a group. For example, if an index used positive scoring for a food group (e.g., whole grains are assigned positive scores on the hPDI), participants received a score of 5 if they were in the highest quintile of consumption for the food group, and a score of 1 if they were in the lowest quintile of consumption (including no consumption). If an index used reverse scoring (e.g., whole grains are assigned reverse scores on the uPDI), participants received a score of 1 if they were in the highest quintile of consumption, and a score of 5 if they were in the lowest quintile or did not consume foods in that grouping. For each participant, scores for the 18 food groups were totaled to obtain their index-specific score. All the plant-based indices have a theoretical range from 18 to 90 with higher scores, indicating greater concordance with the dietary index of interest. The observed ranges for the PDI, hPDI, and uPDI scores were 32 to 79, 31 to 81, and 27 to 77, respectively.

The scores from the indices were operationalized in two approaches: a baseline score and a time-dependent cumulative average score. The baseline score used only the first dietary measurement. This approach allowed us to assess diet around the time of diagnosis. The cumulative average score used the time-updated average of all scores if a participant had repeat dietary measurements. Baseline dietary measurements were carried forward for participants missing follow-up questionnaires, thus assuming dietary intake to be constant over the study period. Using time-dependent cumulative average scores allowed us to account for long-term diet.

### 2.3. Ascertainment of Breast Cancer Recurrence and Survival

Breast cancer recurrences were identified using a combination of follow-up health status questionnaires and KPNC electronic medical record searches [11]. Breast cancer survival was defined by three outcomes: all-cause, breast-cancer-specific, and non-breast-cancer mortality. Mortality and causes of death were ascertained from KPNC’s Virtual Data Warehouse (VDW) mortality files, which incorporate internal data from the KPNC health system, and external linkages with mortality information from the State of California, the Social Security Administration, and the National Death Index [12]. 

### 2.4. Covariate Selection

Demographic and behavioral covariates collected at the baseline interview included age at diagnosis, race/ethnicity (White, Hispanic, Black, Asian/Pacific Islander, and American Indian/Alaska Native), education (at least high school, some college, college graduate, postgraduate), menopausal status, smoking status (never, former, current), total energy in kcal/d, and physical activity as metabolic equivalent of task hours/week of moderate-vigorous activity (MET-hours/week). Breast cancer diagnosis characteristics including stage at diagnosis, estrogen receptor status (ER), and human epidermal growth factor receptor 2 status (HER2) were obtained from the VDW tumor file, which is based on data from the KPNC Cancer Registry [12]. The KPNC Cancer Registry meets the standards of the NCI SEER Program and reports to the San Francisco Bay Area and Greater California SEER Registries [13]. 

### 2.5. Statistical Analysis

Spearman correlation coefficients were estimated to compare the scores of PDI, hPDI, and uPDI. Cox proportional hazards models were used to estimate hazard ratios (HR) and 95% confidence intervals (CI) for recurrence, all-cause mortality, breast-cancer-specific mortality, and non-breast-cancer mortality. Separate models were fit for PDI, hPDI, and uPDI. Within the regression analyses, all scores were expressed as a 10-point continuous scale. Expressing the score as quintiles and restricted cubic splines was considered, but we found no evidence of non-linearity, and the 10-point continuous scale was the selected method using the Bayesian Information Criterion. Person-time was calculated as the years from baseline dietary assessment to the date of first confirmed recurrence or death, depending on the model. If no event occurred, participants were censored at the end of the study period, 31 December 2018. 

Two models for each plant-based index were fit. Model 1 is a minimally adjusted model that adjusted for age at diagnosis, total energy intake in kcal, and physical activity in MET-hours/week. Model 2 is a stratified multivariable-adjusted model that adjusted for covariates in Model 1, and additionally for education, race/ethnicity, smoking status, menopausal status, HER2 status, and stratified by tumor stage and ER status (Model 2). 

All statistical analyses were conducted using R [14]. Specifically, the survival analyses were enabled by the survival package, tables were generated using gtsummary and flextable packages, and figures were generated using ggplot2, gridExtra, and patchwork packages [15,16,17,18,19,20].

## 3. Results

### 3.1. Study Population

The mean age at diagnosis was 60 years (SD, 12 years). Baseline characteristics of the study population stratified by PDI quintiles are listed in Table 1. The baseline characteristics stratified by hPDI and uPDI are listed in Tables S2 and S3, respectively. Before the 10-point rescaling of the indices scores, the average index scores were 53.96 (PDI, SD: 6.72), 54.22 (hPDI, SD: 8.19), and 53.78 (uPDI, SD: 8.19). Cohort-specific quintile cut points are reported within Table 1 for PDI, Table S2 for hPDI, and Table S3 for uPDI, respectively. Due to the scoring methodology, hPDI and uPDI are perfectly inversely correlated (correlation coefficient of −1). Neither index was strongly correlated with PDI (hPDI r = 0.16; *p*-value < 0.001 and uPDI r = −0.16; *p*-value < 0.001). A total of 461 breast cancer recurrences and 653 deaths occurred during a median follow-up period of 9.2 years for recurrence and 9.51 years for deaths (range: 0.05 to 12.9 years). A higher proportion of participants with repeated measurements were white, had a post-graduate education, and were postmenopausal. 

### 3.2. Food Consumption Patterns

**Figure 1:**
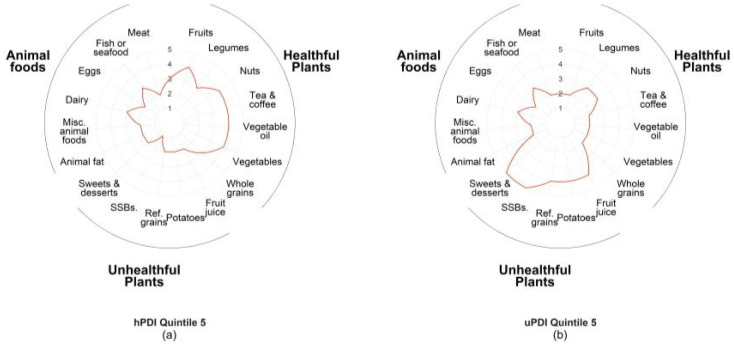
Food groups contributing to plant-based diet indices among breast cancer survivors in the Pathways Study: (**a**) radar plot showing the food consumption patterns of the highest quintile and greatest concordance with hPDI (healthful plant-based diet index). The line represents the median score for each food group within the quintile. A higher score indicates greater consumption of the food group; (**b**) radar plot showing the food consumption patterns of the highest quintile of uPDI (unhealthful plant-based diet index). The line represents the median score for each food group within the quintile. A higher score indicates greater consumption of the food group.

In Figure 1, we used radar plots to qualitatively assess the overall food consumption patterns of participants with the greatest concordance (quintile 5) with hPDI (Figure 1a) and greatest concordance (quintile 5) with uPDI (Figure 1b). In both radar plots, the line represents the median score assigned for each food group for the quintile. If the line is pulled toward the outside of the circle, the median score is higher, indicating greater consumption of that food group. Overall, to achieve high concordance with hPDI, participants did not exclude animal foods or unhealthful plants from their diets but consumed low amounts in each of these food categories (median points 1–3). However, the preponderance of their dietary intake was healthful plants (e.g., whole grains, vegetables, and legumes). This trend is similar in the participants with a high concordance of uPDI, in which the median score reveals a low to moderate consumption of animal foods and healthful plants, but their diet was skewed toward unhealthful plants (e.g., sweets and desserts, potatoes, refined grains, fruit juices and sugar-sweetened beverages (SSBs)).

### 3.3. Plant-Based Indices and Breast Cancer Recurrence and Survival

**Figure 2:**
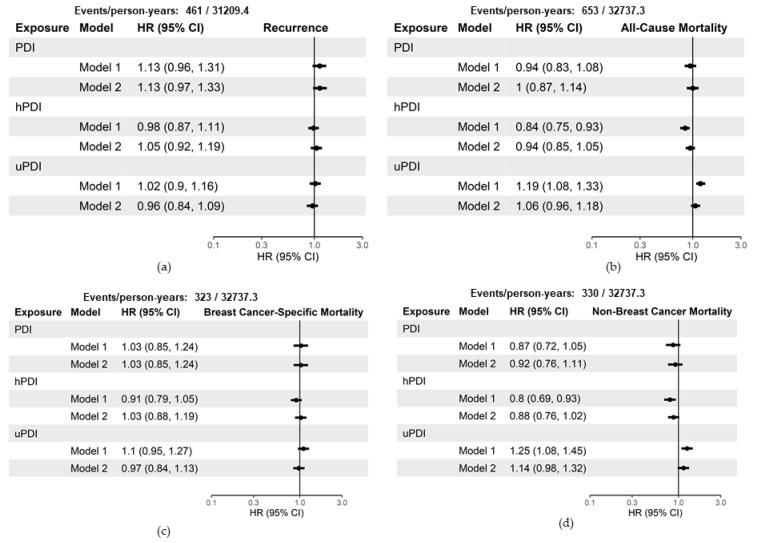
Hazard ratios and 95% confidence intervals for a 10-unit increase in baseline measurements of plant-based indices with breast cancer recurrence and survival among 3646 breast cancer survivors, Pathways Study. Model 1 adjusted for the following covariates: age at diagnosis, total energy intake (kcal/d), and physical activity (moderate-vigorous MET-hours/week). Model 2 adjusted for the following covariates: Model 1 covariates, race/ethnicity, education, menopausal status, smoking status, and stratified by tumor stage and ER status: (**a**) includes estimates of the hazard of recurrence; (**b**) estimates of the hazard of all-cause mortality; (**c**) estimates of the hazard of breast-cancer-specific mortality; (**d**) estimates of the hazard of non-breast-cancer mortality. CI = confidence interval; HR = hazard ratio; PDI = plant-based diet index; hPDI = healthful plant-based diet index; uPDI = unhealthful plant-based diet index.

Figure 2 shows the hazard ratios and 95% confidence intervals for a 10-unit increase in baseline scores. When using the baseline scores in Model 1, a 10-point increase in concordance with hPDI has a statistically significant inverse relationship with all-cause mortality (HR 0.84, 95% CI: 0.75–0.93) and non-breast-cancer mortality (HR 0.80, 95% CI: 0.69–0.93). Conversely, a 10-point increase in concordance with uPDI has a statistically significant positive relationship with all-cause mortality (HR 1.19, 95% CI 1.08–1.33) and non-breast-cancer mortality (HR 1.25, 95% CI: 1.08–1.45). When adjusting for additional demographic characteristics and stratifying on tumor characteristics in Model 2, the estimates are no longer statistically significant for all-cause mortality (hPDI HR 0.94, 95% CI: 0.85–1.05; uPDI HR 1.06, 95% CI: 0.96–1.18) and non-breast-cancer mortality (hPDI HR 0.88, 95% CI: 0.88, 0.76–1.02; uPDI HR 1.14, 95% CI: 0.98–1.32).

**Figure 3:**
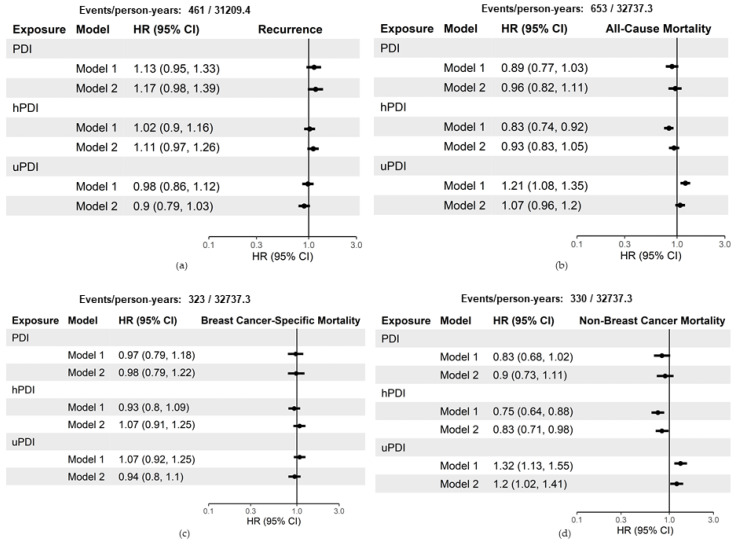
Hazard ratios and 95% confidence intervals for a 10-unit increase in time-dependent cumulative average plant-based indices with breast cancer recurrence and survival amongst 3646 breast cancer survivors, Pathways Study. Model 1 adjusted for the following covariates: age at diagnosis, total energy intake (kcal/d), and physical activity (moderate-vigorous MET-hours/week). Model 2 adjusted for the following covariates: Model 1 covariates, race/ethnicity, education, menopausal status, smoking status, and stratified by tumor stage and ER status: (**a**) includes estimates of the hazard of recurrence; (**b**) estimates of the hazard of all-cause mortality; (**c**) estimates of the hazard of breast-cancer-specific mortality; (**d**) estimates of the hazard of non-breast-cancer mortality. CI = confidence interval; HR = hazard ratio; PDI = plant-based diet index; hPDI = healthful plant-based diet index; uPDI = unhealthful plant-based diet index.

Figure 3 shows the hazard ratios and 95% confidence intervals for a 10-unit increase in the time-dependent cumulative average scores. When using the time-dependent cumulative average score in Model 1, models results were consistent with the baseline scores. hPDI had an inverse relationship and uPDI had a positive relationship with all-cause mortality and non-breast-cancer mortality. In Model 2, a 10-point increase in concordance with hPDI had no association with all-cause mortality (HR 0.93, 95% CI: 0.83, 1.05) and a reduced hazard of non-breast-cancer mortality (HR 0.83, 95% CI: 0.71–0.98). In contrast, a 10-point increase in concordance with uPDI had no association with all-cause mortality (HR 1.07, 95% CI: 0.96–1.2) and an increased hazard of non-breast-cancer mortality (HR 1.20, 95% CI: 1.02–1.41). A 10-point increase in concordance with PDI had no association with all-cause mortality (HR 0.96, 95% CI: 0.82–1.11) and non-breast-cancer mortality (HR 0.90, 95% CI: 0.73–1.11). 

Neither PDI, hPDI, and uPDI were associated with recurrence or breast cancer mortality, regardless of exposure method (baseline vs. time-dependent cumulative average) or model (Model 1 vs. Model 2) (Figure 2 and Figure 3).

## 4. Discussion

In this study of 3646 breast cancer survivors, we found that concordance with healthful plant-based eating patterns (hPDI) in the postdiagnosis period (time-dependent cumulative measurements) reduced the risk of non-breast-cancer mortality. In contrast, greater concordance with unhealthful plant-based eating patterns (uPDI) increased non-breast-cancer mortality risk. We observed no associations between plant-based eating patterns and breast cancer recurrence or breast-cancer-specific mortality. 

Few studies have addressed long-term dietary patterns and prognosis after breast cancer, and none have examined the hPDI, uPDI, or PDI. Even fewer studies have examined dietary patterns and breast cancer recurrence, and none emphasized the distinction between healthful and unhealthful plant foods or found associations with recurrence [21]. Several cohorts have examined other popular a priori dietary pattern indices that are not necessarily “plant based” but do emphasize healthful plant foods such as fruits, legumes, and whole grains, e.g., healthy eating index (HEI), dietary approaches to stop hypertension (DASH), and alternative eating index (AHEI) [21,22,23,24]. Consistent with our findings, a majority of these studies have found protective associations of dietary patterns emphasizing healthful plant foods with all-cause mortality and non-breast-cancer mortality [23,25]. 

Differentiating between healthful and unhealthful plants in plant-based diet indices is a relatively new concept [7]. Our findings of higher hPDI scores being associated with reduced all-cause mortality in the minimally adjusted model and associated with reduced non-breast-cancer mortality in the fully adjusted model and higher uPDI scores being associated with increased mortality are consistent with a study using National Health and Nutrition Examination Survey (NHANES) III [26]. In this study, a 10-unit increase above the median hPDI was associated with a reduced hazard of all-cause mortality in their study population [26]. However, this study observed no association between uPDI and all-cause mortality. In the Nurses’ Health Study (NHS) and NHS2 cohorts, an inverse relationship between hPDI and coronary heart disease, and a positive relationship between uPDI and coronary heart disease were observed [7]. Our findings are consistent with these observations as cardiovascular disease is a major cause of death among breast cancer survivors [27]. 

Though prior research has shown healthful dietary patterns to be important for breast cancer prevention, our study did not observe an association between hPDI, uPDI, and PDI and recurrence or mortality after a breast cancer diagnosis. Potential explanations include that the effect of plant-based diets on breast-cancer-specific outcomes, if they exist, are small, and overwhelmed by the strong influence of breast tumor characteristics (e.g., stage and hormone receptor status) for which we controlled. Another possibility is that a precision nutrition approach with specific foods or nutrients, rather than overall concordance with a healthful, plant-based diet, would be influential in specific breast cancer subtypes [2]. Further, while our median follow-up was 9 years, many breast cancers recur more than a decade after initial diagnosis; it is possible long-term adherence to healthful, plant-based dietary patterns could impact later recurrences or second cancers [28]. 

No prior study has examined plant-based diets and breast-cancer-specific outcomes such as recurrence, making direct comparison difficult, and previous studies of breast cancer incidence have had inconsistent results [8,9,10]. In the Seguimiento Universidad de Navarra cohort (SUN), they observed an inverse association between a healthful version of the pro-vegetarian diet and breast cancer incidence and a positive association between an unhealthful pro-vegetarian diet and breast cancer incidence; however, neither finding was statistically significant [8]. Other case–control studies had differing results between hPDI, uPDI, and risk of breast cancer [9,10]. 

Our study assessed plant-based dietary indices using baseline and time-dependent cumulative average scores. We used both approaches to assess the effect of concordance with plant-based diets at diagnosis and in the postdiagnosis period. We observed that the cumulative average scores, as compared to the baseline scores, yielded stronger associations between hPDI and uPDI, and non-breast-cancer mortality. One explanation for these differences is that an individual’s long-term maintenance of a dietary pattern matters more with regard to breast cancer survival than their diet at the time of diagnosis. However, it is also possible that leveraging repeated measures of a diet (rather than a one-time dietary measurement at baseline) mitigates measurement error, resulting in stronger associations. In addition, since we were observing usual consumption patterns, we could not assess the impact of maximum adherence to any variation of a plant-based diet, as no participant in our study scored at the theoretical maximum. A greater contrast in extremes of exposure could be achieved through a dietary intervention that coaches or provides food to help participants achieve high levels of concordance.

There are several strengths to this study, including its population being a large, representative prospective breast cancer survivor cohort recruited from a community setting. By combining active data collection via study questionnaires with KPNC electronic medical records and state and federal data sources, we were able to leverage a rich resource of covariates and outcomes data. With a long follow-up period and repeated measures, we were able to (1) have more robust measures of baseline and long-term dietary intake, (2) distinguish between the quality of different plant food patterns, and (3) assess, for the first time, the association between long-term concordance with healthful and unhealthful plant-based diets and breast cancer recurrence and survival. 

## 5. Conclusions

In summary, healthful plant-based dietary patterns may reduce the risk of non-breast-cancer mortality, whereas an unhealthful plant-based dietary pattern may increase the risk of this outcome. It is thus important to consider the quality of plant foods to achieve a healthful dietary pattern. Healthful plant-based dietary patterns may improve overall survival in breast cancer survivors. 

## Figures and Tables

**Table 1:** Baseline characteristics of participants by plant-based diet index (PDI) quintiles, Pathways Study.

	Overall	Quintiles of PDI Score				
Characteristic	*n* = 3646 ^1^	Q1, *n* = 784 ^1^	Q2, *n* = 749 ^1^	Q3, *n* = 815 ^1^	Q4, *n* = 682 ^1^	Q5, *n* = 616 ^1^
		Scores: 32–48	Scores: 49–52	Scores: 53–56	Scores: 57–60	Scores: 61–79
Age at diagnosis	60 (12)	60 (12)	60 (12)	60 (12)	59 (12)	58 (12)
BMI (kg/m^2^)	28 (7)	29 (7)	28 (7)	28 (7)	28 (7)	28 (7)
Physical Activity (MET h/week)	54 (36)	46 (34)	50 (34)	54 (35)	58 (36)	63 (39)
Energy intake (kcal/day)	1465 (568)	1112 (432)	1296 (461)	1481 (505)	1679 (551)	1864 (580)
Race/Ethnicity						
White	2481 (68%)	552 (70%)	501 (67%)	563 (69%)	446 (65%)	419 (68%)
Black	237 (6.5%)	51 (6.5%)	53 (7.1%)	51 (6.3%)	45 (6.6%)	37 (6.0%)
Asian/Pacific Islander	474 (13%)	96 (12%)	96 (13%)	111 (14%)	93 (14%)	78 (13%)
Hispanic	378 (10%)	74 (9.4%)	85 (11%)	73 (9.0%)	77 (11%)	69 (11%)
American Indian/Alaska Native	76 (2.1%)	11 (1.4%)	14 (1.9%)	17 (2.1%)	21 (3.1%)	13 (2.1%)
Education						
High school or less	544 (15%)	138 (18%)	129 (17%)	118 (14%)	85 (12%)	74 (12%)
Some college	1241 (34%)	305 (39%)	239 (32%)	269 (33%)	241 (35%)	187 (30%)
College graduate	1022 (28%)	194 (25%)	223 (30%)	226 (28%)	199 (29%)	180 (29%)
Postgraduate	839 (23%)	147 (19%)	158 (21%)	202 (25%)	157 (23%)	175 (28%)
Menopausal Status						
Premenopausal	1057 (29%)	207 (26%)	205 (27%)	236 (29%)	207 (30%)	202 (33%)
Postmenopausal	2589 (71%)	577 (74%)	544 (73%)	579 (71%)	475 (70%)	414 (67%)
Smoking status						
Never	2091 (57%)	422 (54%)	410 (55%)	469 (58%)	418 (61%)	372 (60%)
Former	1403 (38%)	325 (41%)	305 (41%)	317 (39%)	233 (34%)	223 (36%)
Current	152 (4.2%)	37 (4.7%)	34 (4.5%)	29 (3.6%)	31 (4.5%)	21 (3.4%)
AJCC Cancer Stage						
1	1998 (55%)	424 (54%)	411 (55%)	463 (57%)	369 (54%)	331 (54%)
2	1247 (34%)	279 (36%)	267 (36%)	268 (33%)	226 (33%)	207 (34%)
3	346 (9.5%)	72 (9.2%)	60 (8.0%)	69 (8.5%)	77 (11%)	68 (11%)
4	55 (1.5%)	9 (1.1%)	11 (1.5%)	15 (1.8%)	10 (1.5%)	10 (1.6%)
ER Status						
Positive	3063 (84%)	651 (83%)	631 (84%)	680 (83%)	573 (84%)	528 (86%)
Negative	583 (16%)	133 (17%)	118 (16%)	135 (17%)	109 (16%)	88 (14%)
HER2 Status						
Positive	469 (13%)	103 (13%)	92 (12%)	109 (13%)	86 (13%)	79 (13%)
Negative	3037 (83%)	654 (83%)	628 (84%)	673 (83%)	567 (83%)	515 (84%)
Missing	140 (3.8%)	27 (3.4%)	29 (3.9%)	33 (4.0%)	29 (4.3%)	22 (3.6%)

^1^ Mean (SD); *n* (%). PDI = plant-based diet index; BMI = body mass index; MET (h/week) = metabolic equivalent of task hours/week; ER = estrogen receptor; HER2 = human epidermal growth factor receptor 2.

## Data Availability

The data that support the findings of this study are available on request from the corresponding author (E.M.C.F). The data for this study is not publicly available due to it containing protected health information.

## Supplementary Materials

The following are available online at https://www.mdpi.com/article/10.3390/nu13103374/s1, Table S1: Scoring methods and examples of food items comprising the 18 food groups that contribute to PDI, hPDI, and uPDI scores, Table S2: Baseline characteristics of participants by healthful plant-based diet index (hPDI) quintiles, Pathways Study, Table S3: Baseline characteristics of participants by unhealthful plant-based diet index (uPDI) quintiles, Pathways Study.
