# Supplementary material for: Plant-Based Dietary Patterns and Breast Cancer Recurrence and Survival in the Pathways Study
Source: Nutrients. 2021 Sep 25;13(10):3374. doi: 10.3390/nu13103374 (PMC8540603; doi:10.3390/nu13103374)
Supplement: Supplementary file 1 [file nutrients-13-03374-s001.zip › nutrients-1328085-supplementary.pdf]

Anyene et al. Plant-based dietary patterns and breast cancer recurrence and survival in the Pathways Study

Supplementary Materials

Table S1: Scoring methods and examples of food items comprising the 18 food groups that contribute to PDI, hPDI, and uPDI scores.

**Food Groups**	**Example food descriptions**	*Scoring Method*		
		**PDI**	**hPDI**	**uPDI**
**Healthful Plant Foods**				
Whole grains	Whole grains such as breakfast cereal, brown rice, and oatmeal	Positive	Positive	Reverse
Fruits	Whole fruits such as bananas, apples, oranges, and blueberries	Positive	Positive	Reverse
Vegetables	Dark green, red-orange, and starchy vegetables such as tomatoes, broccoli, carrots, and yams	Positive	Positive	Reverse
Nuts	Peanuts, tree nuts, and seeds	Positive	Positive	Reverse
Legumes	Legumes such as beans, lentils, and peas	Positive	Positive	Reverse
Vegetable oils	Salad dressing: oil and vinegar, canola oil, olive oil, and Crisco	Positive	Positive	Reverse
Tea & Coffee	Regular coffee, decaf coffee; green, black, and herbal teas	Positive	Positive	Reverse
**Unhealthful Plant Food**				
Fruit juices	Citrus and non-citrus fruit juices such as orange juice and apple juice	Positive	Reverse	Positive
Refined grains	Refined grains such as bagels, muffins, white rice, and white bread	Positive	Reverse	Positive
Potatoes	White potatoes in forms such as french fries, baked potatoes, etc.	Positive	Reverse	Positive
Sugar-sweetened beverages	Sodas, carbonated beverages with sugar, etc.	Positive	Reverse	Positive
Sweets and desserts	Sweet beverages (excl. juice), pancakes, pastries, and desserts	Positive	Reverse	Positive
**Animal Food**				
Animal fat	Products such as butter, ghee, margarine, and bacon fat	Reverse	Reverse	Reverse
Dairy	Yogurt, ice cream, whole milk, cheese, etc.	Reverse	Reverse	Reverse
Egg	Eggs and egg substitutes	Reverse	Reverse	Reverse
Fish or Seafood	Finfish, shellfish, shrimp, and other seafood	Reverse	Reverse	Reverse
Meat	Chicken, turkey, Cornish hens, duck, goose, processed meats, etc.	Reverse	Reverse	Reverse
Misc. animal-based foods	Pizza, cream soups like chowders, mayonnaise, and sandwich spreads	Reverse	Reverse	Reverse

PDI, hPDI, and uPDI are derived using the 18 food groups listed above. A participant's total serving size consumption for each food group is broken into cohort-specific quintiles, and each quintile is given a score between 1 and 5. Each food group uses positive or reverse scores depending on the index of interest.For positive scores, those in the highest cohort-specific quintile for consumption receive a score of 5 and in the lowest receive a score of 1. For reverse scores, those in the highest cohort-specific quintile for consumption receive a score of 1. A participant's total score for each index is calculated by summing up their scores across food groups. This is adapted from the methodology defined by Satija et al (2016).

Reference:

Satija, A., Bhupathiraju, S.N., Rimm, E.B., Spiegelman, D., Chiuve, S.E., Borgi, L., Willett, W.C., Manson, J.E., Sun, Q., Hu, F.B. Plant-Based Dietary Patterns and Incidence of Type 2 Diabetes in US Men and Women: Results from Three Prospective Cohort Studies. *PLoS medicine* **2016**, *13*, e1002039, doi:10.1371/journal.pmed.1002039.

Anyene et al. Plant-based dietary patterns and breast cancer recurrence and survival in the Pathways Study

Table S2. Baseline characteristics of participants by healthful plant-based diet index (hPDI) quintiles, Pathways Study.

	Overall	Quintiles of hPDI score				
Characteristic	N = 3,646[1]	Q1, N = 799[1] *Scores: 31-47*	Q2, N = 734[1] *Scores: 48-52*	Q3, N = 675[1] *Scores: 53-56*	Q4, N = 728[1] *Scores: 57-61*	Q5, N = 710[1] *Scores: 62-81*
**Age at diagnosis**	60 (12)	58 (12)	59 (12)	60 (12)	61 (11)	61 (11)
**BMI (kg/m2)**	28 (7)	30 (8)	29 (7)	29 (7)	28 (6)	27 (6)
**Physical Activity (MET h/wk)**	54 (36)	46 (31)	52 (36)	54 (34)	52 (34)	65 (41)
**Energy intake (kcal/d)**	1,465 (568)	1,786 (629)	1,506 (522)	1,398 (536)	1,345 (514)	1,250 (451)
**Race/Ethnicity**						
White	2,481 (68%)	487 (61%)	489 (67%)	471 (70%)	527 (72%)	507 (71%)
Black	237 (6.5%)	105 (13%)	38 (5.2%)	35 (5.2%)	26 (3.6%)	33 (4.6%)
Asian/ Pacific Islander	474 (13%)	106 (13%)	102 (14%)	76 (11%)	90 (12%)	100 (14%)
Hispanic	378 (10%)	82 (10%)	86 (12%)	77 (11%)	70 (9.6%)	63 (8.9%)
American Indian/ Alaska Native	76 (2.1%)	19 (2.4%)	19 (2.6%)	16 (2.4%)	15 (2.1%)	7 (1.0%)
**Education**						
High school or less	544 (15%)	151 (19%)	112 (15%)	103 (15%)	92 (13%)	86 (12%)
Some college	1,241 (34%)	309 (39%)	269 (37%)	232 (34%)	228 (31%)	203 (29%)
College graduate	1,022 (28%)	226 (28%)	193 (26%)	194 (29%)	203 (28%)	206 (29%)
Postgraduate	839 (23%)	113 (14%)	160 (22%)	146 (22%)	205 (28%)	215 (30%)
**Menopausal Status**						
Premenopausal	1,057 (29%)	283 (35%)	234 (32%)	198 (29%)	176 (24%)	166 (23%)
Postmenopausal	2,589 (71%)	516 (65%)	500 (68%)	477 (71%)	552 (76%)	544 (77%)
**Smoking status**						
Never	2,091 (57%)	433 (54%)	421 (57%)	403 (60%)	424 (58%)	410 (58%)
Former	1,403 (38%)	309 (39%)	289 (39%)	239 (35%)	279 (38%)	287 (40%)
Current	152 (4.2%)	57 (7.1%)	24 (3.3%)	33 (4.9%)	25 (3.4%)	13 (1.8%)
**AJCC Cancer Stage**						
1	1,998 (55%)	402 (50%)	393 (54%)	342 (51%)	433 (59%)	428 (60%)
2	1,247 (34%)	291 (36%)	252 (34%)	256 (38%)	236 (32%)	212 (30%)
3	346 (9.5%)	93 (12%)	70 (9.5%)	67 (9.9%)	55 (7.6%)	61 (8.6%)
4	55 (1.5%)	13 (1.6%)	19 (2.6%)	10 (1.5%)	4 (0.5%)	9 (1.3%)
**ER Status**						
Positive	3,063 (84%)	654 (82%)	614 (84%)	571 (85%)	626 (86%)	598 (84%)
Negative	583 (16%)	145 (18%)	120 (16%)	104 (15%)	102 (14%)	112 (16%)
**HER2 Status**						
Positive	469 (13%)	116 (15%)	88 (12%)	88 (13%)	89 (12%)	88 (12%)
Negative	3,037 (83%)	662 (83%)	617 (84%)	562 (83%)	611 (84%)	585 (82%)
Missing	140 (3.8%)	21 (2.6%)	29 (4.0%)	25 (3.7%)	28 (3.8%)	37 (5.2%)

[1]Mean (SD); n (%)

hPDI = healthful plant-based diet index; BMI = body mass index; MET (h/wk) = metabolic equivalent of task hours/week; ER = estrogen receptor; HER2 = human epidermal growth factor receptor 2

Table S3. Baseline characteristics of participants by unhealthful plant-based diet index (uPDI) quintiles, Pathways Study.

	Overall	Quintiles of uPDI score				
Characteristic	N = 3,646[1]	Q1, N = 837[1] *Scores: 27-47*	Q2, N = 752[1] *Scores: 48-52*	Q3, N = 687[1] *Scores: 53-56*	Q4, N = 711[1] *Scores: 57-61*	Q5, N = 659[1] *Scores: 62-77*
**Age at diagnosis**	60 (12)	61 (11)	61 (12)	60 (12)	58 (12)	58 (12)
**BMI (kg/m2)**	28 (7)	27 (6)	28 (6)	29 (7)	29 (7)	30 (8)
**Physical Activity (MET h/wk)**	54 (36)	64 (41)	52 (32)	54 (36)	52 (34)	46 (31)
**Energy intake (kcal/d)**	1,465 (568)	1,257 (458)	1,349 (511)	1,417 (535)	1,567 (554)	1,805 (627)
**Race/Ethnicity**						
White	2,481 (68%)	604 (72%)	530 (70%)	495 (72%)	459 (65%)	393 (60%)
Black	237 (6.5%)	35 (4.2%)	33 (4.4%)	28 (4.1%)	48 (6.8%)	93 (14%)
Asian/ Pacific Islander	474 (13%)	116 (14%)	94 (12%)	70 (10%)	105 (15%)	89 (14%)
Hispanic	378 (10%)	74 (8.8%)	75 (10.0%)	81 (12%)	79 (11%)	69 (10%)
American Indian/ Alaska Native	76 (2.1%)	8 (1.0%)	20 (2.7%)	13 (1.9%)	20 (2.8%)	15 (2.3%)
**Education**						
High school or less	544 (15%)	95 (11%)	111 (15%)	94 (14%)	116 (16%)	128 (19%)
Some college	1,241 (34%)	241 (29%)	241 (32%)	251 (37%)	255 (36%)	253 (38%)
College graduate	1,022 (28%)	248 (30%)	200 (27%)	194 (28%)	188 (26%)	192 (29%)
Postgraduate	839 (23%)	253 (30%)	200 (27%)	148 (22%)	152 (21%)	86 (13%)
**Menopausal Status**						
Premenopausal	1,057 (29%)	195 (23%)	192 (26%)	190 (28%)	241 (34%)	239 (36%)
Postmenopausal	2,589 (71%)	642 (77%)	560 (74%)	497 (72%)	470 (66%)	420 (64%)
**Smoking status**						
Never	2,091 (57%)	486 (58%)	431 (57%)	410 (60%)	412 (58%)	352 (53%)
Former	1,403 (38%)	334 (40%)	296 (39%)	244 (36%)	272 (38%)	257 (39%)
Current	152 (4.2%)	17 (2.0%)	25 (3.3%)	33 (4.8%)	27 (3.8%)	50 (7.6%)
**AJCC Cancer Stage**						
1	1,998 (55%)	506 (60%)	433 (58%)	352 (51%)	375 (53%)	332 (50%)
2	1,247 (34%)	247 (30%)	258 (34%)	253 (37%)	251 (35%)	238 (36%)
3	346 (9.5%)	74 (8.8%)	55 (7.3%)	72 (10%)	68 (9.6%)	77 (12%)
4	55 (1.5%)	10 (1.2%)	6 (0.8%)	10 (1.5%)	17 (2.4%)	12 (1.8%)
**ER Status**						
Positive	3,063 (84%)	711 (85%)	637 (85%)	591 (86%)	589 (83%)	535 (81%)
Negative	583 (16%)	126 (15%)	115 (15%)	96 (14%)	122 (17%)	124 (19%)
**HER2 Status**						
Positive	469 (13%)	102 (12%)	90 (12%)	83 (12%)	102 (14%)	92 (14%)
Negative	3,037 (83%)	690 (82%)	638 (85%)	575 (84%)	584 (82%)	550 (83%)
Missing	140 (3.8%)	45 (5.4%)	24 (3.2%)	29 (4.2%)	25 (3.5%)	17 (2.6%)

[1]Mean (SD); n (%)

uPDI = unhealthful plant-based diet index; BMI = body mass index; MET (h/wk) = metabolic equivalent of task hours/week; ER = estrogen receptor; HER2 = human epidermal growth factor receptor 2
